# Significance of Neutrophil-to-Lymphocyte Ratio in Predicting the Efficacy of Anamorelin for Cancer Cachexia

**DOI:** 10.7759/cureus.77795

**Published:** 2025-01-21

**Authors:** Yusuke Nakazawa, Kanako Watanabe, Ako Gannichida, Tadashi Uwagawa, Takashi Kawakubo

**Affiliations:** 1 Department of Pharmacy, The Jikei University Hospital, Tokyo, JPN; 2 Department of Surgery, The Jikei University School of Medicine, Tokyo, JPN

**Keywords:** anamorelin, cancer cachexia, efficacy prediction, neutrophil-to-lymphocyte ratio, prognostic biomarker

## Abstract

Introduction

Cancer cachexia is a multifactorial syndrome characterized by persistent skeletal muscle loss and poor prognosis in cancer patients. Anamorelin, a ghrelin receptor agonist, may alleviate cachexia symptoms by increasing appetite and promoting weight gain, though its clinical efficacy remains insufficiently explored. Given the involvement of cancer-inducing cytokines in cachexia, the neutrophil-to-lymphocyte ratio (NLR), an inflammatory biomarker, may serve as a predictor of therapeutic outcomes in this condition. This study aimed to evaluate the role of NLR in assessing the therapeutic effects of anamorelin and its prognostic value in patients with cancer cachexia.

Methods

This study included patients with cancer cachexia associated with pancreatic, non-small cell lung, gastric, or colorectal cancer who received anamorelin between April 2021 and December 2023. Patients were categorized based on their NLR (<5 or ≥5) at four weeks post-anamorelin administration. Changes in NLR and one-year overall survival (OS) rates were compared between groups. Baseline NLR was also assessed for its impact on survival outcomes. Statistical analyses included the Kaplan-Meier method for survival analysis, and receiver operating characteristic (ROC) analysis was used to determine the optimal baseline NLR cutoff for achieving a posttreatment NLR < 5.

Results

Of the 66 patients who received anamorelin, 42 had pancreatic cancer, 14 had non-small cell lung cancer, 6 had gastric cancer, and 4 had colorectal cancer. Patients were stratified into two groups based on their NLR at four weeks: NLR < 5 (n = 50, 76%) and NLR ≥ 5 (n = 16, 24%). In the NLR < 5 group, the mean NLR decreased significantly from 3.71 to 2.44, while in the NLR ≥ 5 group, it increased from 5.70 to 9.52. The one-year OS was significantly higher in the NLR < 5 group (62%, n = 31/50) compared to the NLR ≥ 5 group (33%, n = 5/16). Baseline NLR classification, however, showed no significant survival difference between the baseline NLR < 5 group (58%, n = 21/51) and the NLR ≥ 5 group (41%, n = 6/15). ROC analysis identified a baseline NLR < 4.4 as predictive of achieving a posttreatment NLR < 5 (AUC, 0.78; sensitivity, 80%; specificity, 75%), with significantly higher one-year OS observed in the baseline NLR < 4.4 group (68%, n = 29/42) compared to the NLR ≥ 4.4 group (34%, n = 8/24).

Conclusions

These findings highlight the potential of NLR as a prognostic marker and suggest that initiating anamorelin treatment with a baseline NLR < 4.4 is likely to improve outcomes, emphasizing the importance of NLR-based therapeutic interventions.

## Introduction

Cancer cachexia is a multifactorial syndrome characterized by weight loss, muscle weakness, anorexia, fatigue, and mental health issues that are difficult to reverse with conventional nutritional support [1-4]. Early diagnosis and intervention during the reversible stages are crucial, as cachexia in refractory cancer is unlikely to respond to therapeutic interventions [5-9]. In 2021, Japan approved anamorelin as the world’s first treatment for cancer cachexia. Anamorelin has shown promise in alleviating cancer cachexia symptoms by stimulating the ghrelin receptor GHS-R1a [10,11], thereby increasing appetite and promoting weight gain [11-13]. However, while recent studies have explored predictors of response to anamorelin, clinical evidence linking improvements in cancer cachexia with survival outcomes remains limited.

Cancer cachexia is driven by fat and muscle degradation, metabolic abnormalities, and appetite suppression, mediated by cancer-host interactions and cancer-induced cytokines [14,15]. Given the involvement of numerous inflammatory cytokines in cancer cachexia, we focused on the neutrophil-to-lymphocyte ratio (NLR), an inflammatory biomarker. Neutrophils increase during inflammation and may promote cytokines production involved in tumor progression, while lymphocytes counteract tumor growth and support immune response. NLR has been recognized as a prognostic marker in oncology [16-18], with a typical cutoff value of NLR < 5, indicating its association with favorable treatment outcomes and prognosis [19-21].

Given the limited data on the prognostic value of anamorelin, this study investigated NLR changes following anamorelin administration and their impact on prognosis, aiming to establish the role of NLR in predicting optimal treatment efficacy.

This article was previously posted on the Research Square preprint server on May 10, 2024.

## Materials and methods

Patient selection

This retrospective study included patients receiving anamorelin for cancer cachexia associated with pancreatic, non-small cell lung, gastric, and colorectal cancers, for which indications are approved in Japan, at The Jikei University Hospital (3-19-18 Nishi Shimbashi, Minato-ku, Tokyo 105-8471, Japan) between April 2021 and December 2023. Patients were identified through electronic medical records. Inclusion criteria were patients diagnosed with cancer cachexia and those undergoing chemotherapy for advanced cancers. Patients with a performance status of ≥3, nonresponse to anticancer therapy, and death within three months of starting anamorelin administration were excluded, as they were considered to have refractory cancer cachexia and were unlikely to benefit from anamorelin treatment.

Data collection

Patient data, including demographic characteristics, cancer type, laboratory results, and survival outcomes, were extracted from electronic medical records. Key variables included baseline NLR and NLR at four weeks post-anamorelin treatment. Follow-up data were collected four weeks after treatment, and survival outcomes were assessed over a one-year period from the initiation of anamorelin treatment. Previous studies have shown that the primary effects of anamorelin, such as weight gain and improved appetite, typically manifest within four weeks of administration. This evaluation period was set at four weeks after the administration of anamorelin [22]. This evaluation period also aligns with the standard four-week chemotherapy cycle at our hospital.

Group classification and survival analysis

Anamorelin was administered at a daily dose of 100 mg. To evaluate the variability in NLR during anamorelin treatment, patients were categorized into two groups based on their NLR at four weeks after treatment: NLR < 5 and NLR ≥ 5. The NLR cutoff value of 5 was chosen based on previous studies [19-21]. Changes in baseline NLR and NLR at four weeks after treatment were analyzed within each group. Overall survival (OS) was defined as the time from the initiation of anamorelin treatment to the date of death from any cause or the end of the follow-up period. Additionally, to assess the impact of baseline NLR (<5 or ≥5) on survival outcomes, patients were further stratified into two groups based on their baseline NLR: NLR < 5 and NLR ≥ 5. Changes in baseline NLR and NLR at four weeks after treatment, as well as the one-year OS rate, were compared between these groups.

Statistics

The distributions of continuous variables were evaluated using the Shapiro-Wilk test. For comparisons of unpaired continuous variables, the t-test or Mann-Whitney U test was applied based on data distribution. Paired continuous variables were compared using the t-test or Wilcoxon signed-rank test. Survival outcomes were evaluated using the Kaplan-Meier method and compared with the log-rank test. Receiver operating characteristic (ROC) analysis was used to determine the optimal NLR cutoff value predicting an NLR < 5 at four weeks. Logistic regression analysis was performed to assess the association between NLR at treatment initiation and achieving NLR < 5 posttreatment, with the objective variable being the NLR < 5 four weeks posttreatment and the explanatory variable being the patient’s baseline characteristics at treatment initiation. All statistical analyses were conducted using BellCurve for Excel (Social Survey Research Information Co, Ltd, Tokyo, Japan), with statistical significance set at p < 0.05.

Ethics approval

All procedures in this study were conducted in accordance with the ethical standards of the institutional and/or national research committee, as well as the 1964 Helsinki Declaration and its later amendments or comparable ethical standards. This study was approved by the Ethics Committee of Jikei University (Approval No. 34-334(11488)). Informed consent for study participation and data usage was obtained from all patients through an opt-out approach.

## Results

Comparison of patients' background data

Of the 79 patients screened, 66 met the inclusion criteria: 42 with pancreatic cancer, 14 with non-small cell lung cancer, 6 with gastric cancer, and 4 with colorectal cancer. Thirteen patients with refractory cancer cachexia were excluded. Patients were stratified into two groups based on their NLR at four weeks: NLR < 5 (n = 50, 76%) and NLR ≥ 5 (n = 16, 24%). Baseline patient data before the initiation of anamorelin treatment are presented in Table 1. Cancer types in patients with NLR < 5 and ≥ 5 at four weeks after anamorelin treatment were as follows: pancreatic cancer in 33 vs. 9 patients, non-small cell lung cancer in 9 vs. 5 patients, gastric cancer in 6 vs. 0 patients, and colorectal cancer in 2 vs. 2 patients. Laboratory results at four weeks after anamorelin treatment showed mean serum albumin levels of 3.54 ± 0.53 and 3.23 ± 0.34 g/dL, mean lymphocyte counts of 1,406 ± 687 and 988 ± 424 /µL, and mean NLR of 3.71 ± 3.12 and 5.70 ± 2.78, respectively. Patients with NLR < 5 four weeks after treatment had higher albumin levels, higher lymphocyte counts, and lower NLR.

**Table 1:** Patients’ background characteristics before anamorelin treatment. a) Fisher’s exact test, b) Mann–Whitney U test. AST: aspartate aminotransferase, ALT: alanine aminotransferase, CRP: C-reactive protein, WBC: white blood cell, NLR: neutrophil-to-lymphocyte ratio.

Variables	NLR four weeks after		p-value	
	<5 (n = 50)	≥5 (n = 16)		
Male/female	29/21	9/7	1.000	^a)^
Age (year)	70.5 ± 9.3	70.4 ± 9.9	0.952	^b)^
Body weight (kg)	50.2 ± 9.2	49.2 ± 13.9	0.312	^b)^
Cancer type				
Pancreatic cancer	33	9	0.556	^a)^
Non-small cell lung cancer	9	5	0.300	^a)^
Gastric cancer	6	0	0.323	^a)^
Colorectal cancer	2	2	0.245	^a)^
Laboratory value				
AST (U/L)	33.5 ± 33.3	27.8 ± 21.2	0.610	^b)^
ALT (U/L)	30.8 ± 35.3	33.8 ± 70.2	0.166	^b)^
Albumin (g/dL)	3.54 ± 0.53	3.23 ± 0.34	<0.001	^b)^
Albumin/globulin ratio	1.22 ± 0.30	1.06 ± 0.31	0.062	^b)^
Blood urea nitrogen (mg/dL)	16.2 ± 10.4	14.6 ± 5.1	0.691	^b)^
Serum creatinine (mg/dL)	0.78 ± 0.38	0.76 ± 0.24	0.653	^b)^
CRP (mg/dL)	1.43 ± 3.76	2.73 ± 4.78	0.089	^b)^
WBC (/µL)	6,530 ± 5,512	6,844 ± 3,098	0.664	^b)^
Hemoglobin (g/dL)	10.99 ± 1.64	11.23 ± 2.31	0.881	^b)^
Platelet (x 10^3^/µL)	253 ± 133	285 ± 129	0.394	^b)^
Neutrophil count (/µL)	4,518 ± 5,084	5,356 ± 2,947	0.116	^b)^
Lymphocyte count (/µL)	1,406 ± 687	988 ± 424	0.019	^b)^
NLR	3.71 ± 3.12	5.70 ± 2.78	<0.001	^b)^

Fluctuation in NLR four weeks after anamorelin administration

**Figure 1:**
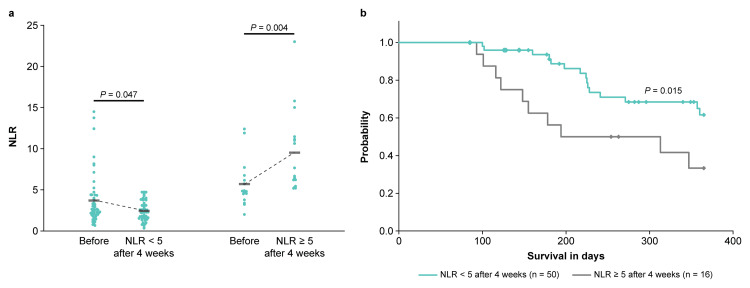
(a) Fluctuation of NLR in patients with NLR < 5 and ≥ 5 four weeks after anamorelin treatment. (b) Overall survival of patients with NLR < 5 and ≥ 5 four weeks after anamorelin treatment. NLR: neutrophil-to-lymphocyte ratio.

The variability in NLR before and after four weeks of anamorelin administration in patients with NLR < 5 and ≥ 5 was compared (Figure 1a). A significant decrease in mean NLR was observed in the NLR < 5 group, from 3.71 ± 3.12 to 2.44 ± 1.23, at four weeks after treatment, while a significant increase in the mean NLR was noted in the NLR ≥ 5 group, from 5.70 ± 2.78 to 9.52 ± 4.85, at four weeks after treatment. Among these patients, 28 (56%) of those with NLR < 5 (n = 50) and 1 (6%) of those with NLR ≥ 5 (n = 16) showed a decrease in NLR at four weeks after treatment, with a significant difference between the two groups (p < 0.001; Fisher’s exact test). Changes in neutrophil and lymphocyte counts (components of NLR) before and after four weeks of anamorelin administration in patients with NLR < 5 and ≥ 5 were compared (Table 2). In the NLR < 5 group, the mean neutrophil count significantly decreased from 4518 ± 5084 to 3036 ± 1903 four weeks after treatment, but no significant difference was observed in the changes in lymphocyte count. On the other hand, in the NLR ≥ 5 group, the mean lymphocyte count significantly decreased from 988 ± 424 to 756 ± 377 at four weeks after treatment, while neutrophil count did not show a significant change. Additionally, at four weeks after treatment, patients with NLR < 5 had a one-year OS rate of 62% (n = 31/50), with the median not reached (95% confidence interval (CI) not reached), while those with NLR ≥ 5 had a one-year OS rate of 33% (n = 5/16), with a median of 194 days (95% CI 0-428 days). This result suggested a significantly higher one-year OS rate among patients with NLR < 5 (p = 0.015; Figure 1b).

**Table 2:** Fluctuation of neutrophil and lymphocyte count after anamorelin treatment. Wilcoxon signed-rank test. NLR: neutrophil-to-lymphocyte ratio.

Variables	NLR < 5 at four weeks after			NLR ≥ 5 at four weeks after		
	Before	At four weeks after	p-value	Before	At four weeks after	p-value
Neutrophil count (/µL)	4518 ± 5084	3036 ± 1903	0.016	5356 ± 2947	6163 ± 2538	0.234
Lymphocyte count (/µL)	1406 ± 687	1326 ± 682	0.157	988 ± 424	756 ± 377	0.046

NLR variability in patients with NLR < 5 or ≥ 5 before anamorelin treatment

**Figure 2:**
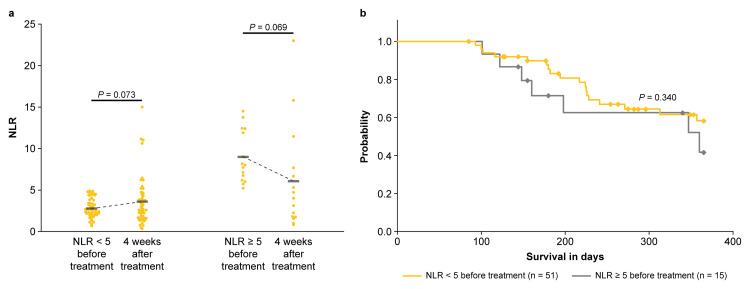
(a) Fluctuation of NLR after four weeks in patients with baseline NLR < 5 and ≥ 5 before anamorelin treatment. (b) Overall survival of patients with baseline NLR < 5 and ≥ 5 before anamorelin treatment. NLR: neutrophil-to-lymphocyte ratio.

NLR variability before and at four weeks after treatment was compared between patients with baseline NLR < 5 and ≥ 5 (Figure 2a). In the baseline NLR < 5 group, the mean NLR ranged from 2.78 ± 1.21 to 3.60 ± 2.92, showing no significant differences. In the NLR ≥ 5 group, the mean NLR ranged from 8.99 ± 3.04 to 6.06 ± 6.08, also showing no significant difference. Among these patients, 19 (37%) in the NLR < 5 group (n = 51) and 10 (67%) in the NLR ≥ 5 group (n = 15) showed a decrease in NLR at four weeks after treatment, with no significant difference between the two groups (p = 0.069; Fisher’s exact test). Furthermore, before the initiation of anamorelin, one-year OS rates for patients with baseline NLR of <5 and ≥5 were 58% (n = 30/51), with the median not reached (95% CI not reached), and 41% (n = 6/15), with a median of 360 days (95% CI 131-589 days), respectively (p = 0.341; Figure 2b).

Cutoff value predicting NLR < 5 at four weeks after anamorelin administration

**Figure 3:**
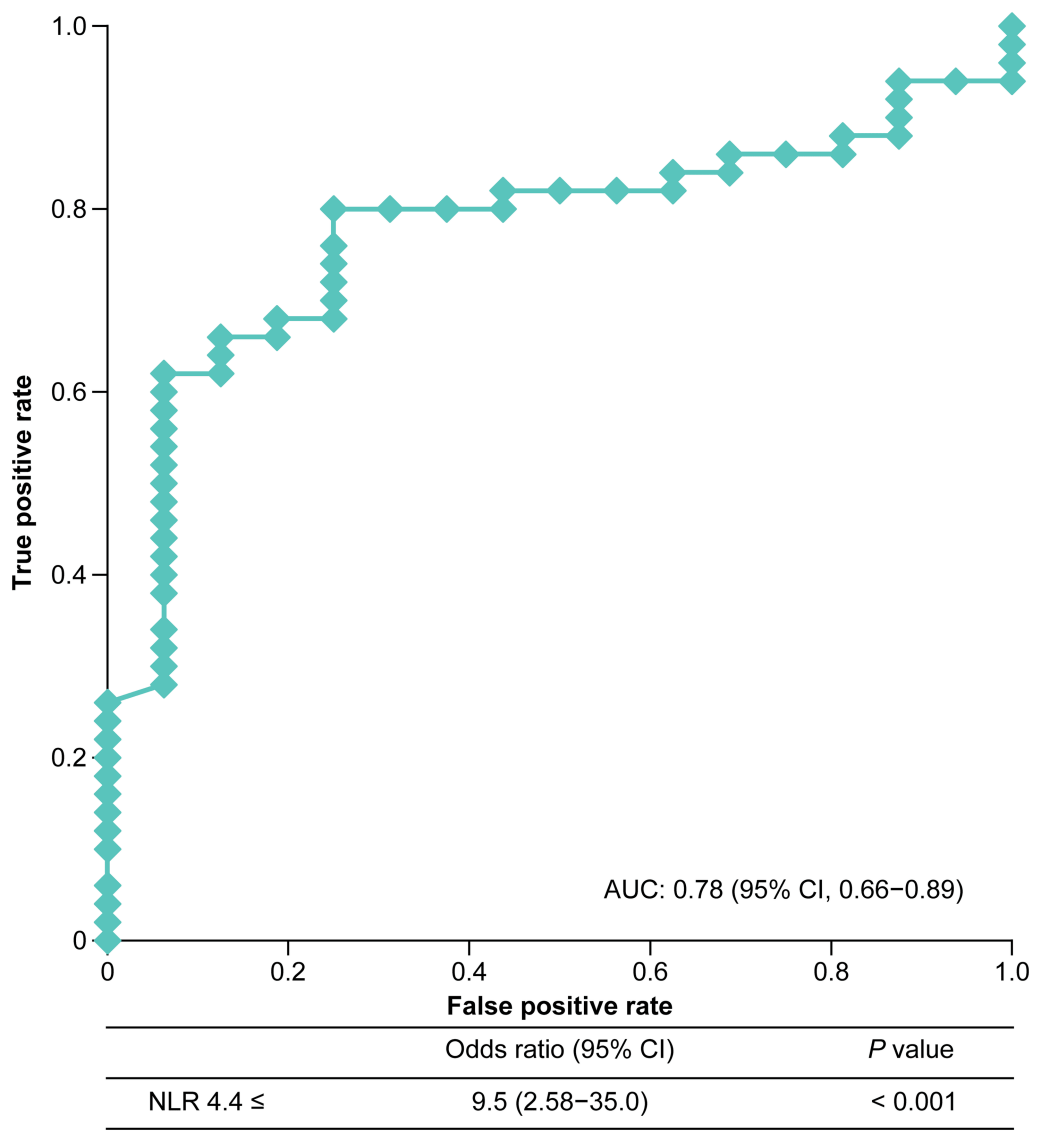
Optimal NLR cutoff value for anamorelin treatment initiation to achieve NLR < 5: receiver operating characteristic analysis. The curve illustrates the tradeoff between the model's sensitivity and specificity. The dots mark the threshold point, yielding a sensitivity of 80% and a specificity of 75%. AUC: area under the receiver operating characteristic curve, CI: confidence interval, NLR: neutrophil-to-lymphocyte ratio.

ROC analysis identified a baseline NLR cutoff value of 4.4 at treatment initiation as predictive of achieving an NLR < 5 at four weeks after treatment, with an area under the curve (AUC) of 0.78, sensitivity of 80%, and specificity of 75% (Figure 3). In the univariate analysis, baseline NLR ≥ 4.4 was a significant risk factor for obtaining an NLR ≥ 5 at four weeks after anamorelin administration (odds ratio, 9.8; 95% CI, 2.58-35.0; p < 0.001).

Logistic regression analysis of factors associated with NLR < 5 after four weeks

**Figure 4:**
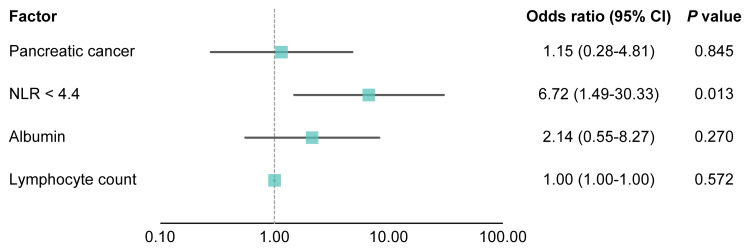
Factors associated with neutrophil-to-lymphocyte ratio (NLR) < 5 at four weeks after by logistic regression analysis. NLR: neutrophil-to-lymphocyte ratio, CI: confidence interval.

Logistic regression analysis was conducted to identify factors associated with achieving an NLR < 5 at 4 weeks after anamorelin initiation. The dependent variable was the presence or absence of NLR < 5 at 4 weeks, while explanatory variables included the presence or absence of pancreatic cancer, baseline NLR < 4.4, albumin level, and lymphocyte count. Figure 4 presents the odds ratios, 95% CIs, and p-values obtained from the univariate logistic regression analysis. Baseline NLR < 4.4 was significantly associated with achieving NLR < 5 at four weeks (p = 0.013).

 Overall survival rates in patients with baseline NLR < 4.4 or ≥ 4.4

**Figure 5:**
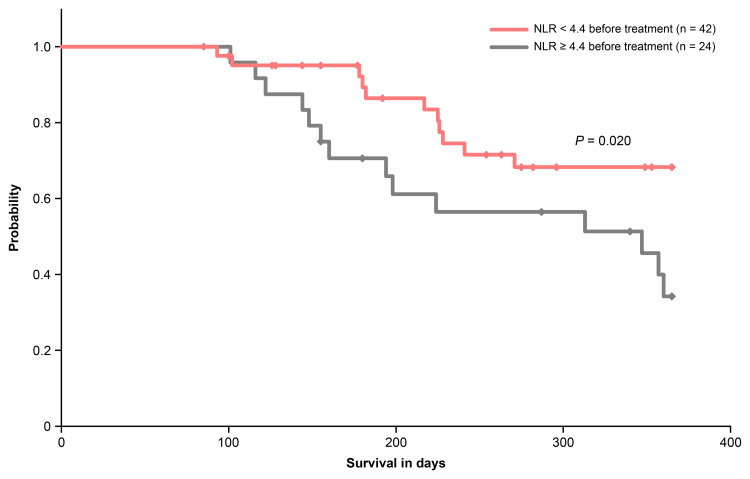
Overall survival of patients with baseline NLR < 4.4 and ≥ 4.4 before anamorelin treatment. NLR: neutrophil-to-lymphocyte ratio.

Based on the cutoff value of 4.4 identified by ROC analysis, one-year OS rates were compared between patients with baseline NLR < 4.4 and ≥ 4.4. The OS rates were 68% (n = 29/42) for those with baseline NLR < 4.4 (median not reached (95% CI not reached)) and 34% (n = 8/24) for those with baseline NLR ≥ 4.4 (median 347 days (95% CI 175-519 days)) (p = 0.020, Figure 5).

## Discussion

Although improving cancer cachexia may enhance quality of life and treatment tolerance, evidence regarding its direct effect on survival outcomes remains insufficient [8]. Anamorelin has demonstrated efficacy in improving appetite and promoting weight gain, though its impact on OS remains unclear [13]. Recent studies suggest that OS may be prolonged in patients who respond to anamorelin [22,23], highlighting the need to identify predictive factors for therapeutic efficacy. Previous reports have indicated that inflammatory markers, such as albumin and C-reactive protein (CRP), could serve as predictors of response in patients who experience improvements in lean body mass following anamorelin treatment [24,25], although definitive conclusions are lacking. One reason for this is the inclusion of patients with refractory cancer cachexia, which may have confounded the results regarding efficacy predictors. The present study aimed to evaluate whether changes in NLR during anamorelin therapy could predict treatment efficacy and prognosis, specifically focusing on patients with reversible cancer cachexia and excluding those with refractory cancer cachexia who were expected to discontinue treatment early. Our results revealed a significant decrease in NLR after four weeks of anamorelin treatment in patients with NLR < 5, along with prolonged one-year OS. To date, no studies have demonstrated anamorelin's effect in lowering NLR. A detailed analysis showed that patients with an NLR < 5 after four weeks of treatment had lower neutrophil counts compared to baseline. Ghrelin, a hormone secreted by the gastric mucosa, is known for its anti-inflammatory properties and appetite-stimulating effects [26]. The reduction in neutrophil count and NLR may have been influenced by the anti-inflammatory effects of anamorelin, a ghrelin receptor agonist. While NLR has been linked to prognosis, a lower NLR does not necessarily indicate better outcomes. Notably, it has been reported that patients with an NLR below a certain threshold of 5 tend to show favorable treatment results [19-21]. Thus, our findings support anamorelin therapy as a promising treatment for cancer cachexia and suggest that achieving an NLR < 5 during treatment may predict better treatment efficacy. Conversely, patients with an NLR ≥ 5 four weeks after treatment showed shorter survival and larger increases in NLR. In these patients, neutrophil counts remained unchanged, while lymphocyte counts decreased. The decrease in lymphocytes may indicate a worsening condition, as lymphocytes play a tumor-suppressive role and serve as indicators of immune function decline [27], which may be associated with a higher NLR and shorter OS.

Furthermore, when comparing the data between patients with baseline NLR < 5 and those with NLR ≥ 5 before anamorelin administration, we found that baseline NLR < 5 was not a predictor of treatment outcome. Instead, achieving an NLR < 5 after treatment proved to be more crucial. While the European Palliative Care Research Collaborative recommends early intervention starting at the pre-cachexia stage [1], management indicators to predict treatment efficacy have yet to be fully established. Therefore, establishing an initial NLR guideline that aims for an NLR < 5 after anamorelin treatment could significantly improve treatment outcomes. Our study demonstrated that a cutoff value of 4.4 may predict an NLR < 5 at four weeks after anamorelin administration. In addition, patients with baseline NLR < 4.4 prior to anamorelin showed significantly longer one-year OS, indicating that the NLR is a useful prognostic marker for survival outcomes. Furthermore, logistic regression analysis also showed that a baseline NLR < 4.4 was significantly associated with achieving an NLR < 5 at four weeks after treatment. However, no significant associations were found between albumin and lymphocyte counts and NLR < 5 after anamorelin administration. Albumin is now recognized as an inflammatory marker; during inflammation, albumin production may decrease due to reduced liver synthesis function and increased capillary permeability [28,29]. Additionally, lymphocytes may be depleted as a result of chronic inflammation, which can cause excessive activation of the immune system [30]. Consequently, these biochemical inflammatory parameters, other than NLR, may serve as important indicators of treatment efficacy and prognosis. Further comprehensive research is required to better understand their significance.

There are several limitations to this study. This was a single-center, retrospective study, and the specific characteristics of cancer treatment modalities and outcomes at the institution were considered. Additionally, the study population included patients with four types of cancer: pancreatic, non-small cell lung, gastric, and colorectal cancers. Since survival duration can vary significantly based on cancer type and stage, detailed analyses for each cancer type are necessary. The limited number of cases for each cancer type suggests the need for further clinical prospective trials and multicenter collaborative studies. Although this study focused on patients with reversible cancer cachexia, there may still be potential for recovery with anamorelin treatment in cases of refractory cancer cachexia. Therefore, further investigation into the effects of anamorelin in patients with irreversible cachexia is warranted.

These findings highlight the utility of monitoring NLR during anamorelin therapy as a prognostic marker and therapeutic target. Achieving an NLR < 5 posttreatment may indicate effective management of cancer cachexia and improved survival outcomes. Furthermore, baseline NLR < 4.4 could serve as a practical guide for initiating therapy, offering a strategy for early intervention.

## Conclusions

NLR is a valuable prognostic marker for assessing anamorelin’s efficacy in cancer cachexia treatment. Monitoring NLR changes and initiating treatment with a baseline NLR < 4.4 may help optimize therapeutic outcomes and improve survival.
